# Supplementary material for: Potential of Lycii Radicis Cortex as an Ameliorative Agent for Skeletal Muscle Atrophy
Source: Pharmaceuticals (Basel). 2024 Apr 4;17(4):462. doi: 10.3390/ph17040462 (PMC11054743; doi:10.3390/ph17040462)
Supplement: Supplementary file 1 [file pharmaceuticals-17-00462-s001.zip › pharmaceuticals-2887918-supplementary.pdf]

Table S1. Primer information.

Sample	Species	Gene	Sequence (F)	Sequence (R)
C2C12 myotubes	Mouse	Myogenin	5’-AGTACATTGAGCGCCTACAG-3’	5’-GACGTAAGGGAGTGCAGATT-3’
	Mouse	Myostatin	5’-CTGTAACCTTCCCAGGACCA-3’	5’-GCAGTCAAGCCCAAAGTCTC-3’
	Mouse	MuRF1	5’-TGCCTACTTGCTCCTTGTGC-3’	5’-CACCAGCATGGAGATGCAGT-3’
	Mouse	Atrogin-1	5’-CTGCCTGTGTGCTTACAACT-3’	5’-TGCTCTCTTCTTGGGTAACA-3’
	Mouse	GAPDH	5’-ACTCCACTCACGGCAAATTC-3’	5’-TCTCCATGGTGGTGAAGACA-3’
Gastrocnemius muscle	Mouse	MuRF1	5’-ACCTGCTGGTGGAAAACATC-3’	5’-AGGAGCAAGTAGGCACCTCA-3’
	Mouse	Atrogin-1	5’-ATGCACACTGGTGCAGAGAG-3’	5’-TGTAAGCACACAGGCAGGTC-3’
	Mouse	IGF-1	5’-GGACCAGAGACCCTTTGCGGGG-3’	5’-GCTGCTTTTGTAGGCTTCAGTGG-3
	Mouse	GAPDH	5’-GTCATCATCTCCGCCCCTTCTGC-3’	5’-GATGCCTGCTCACCACCTTCTTG-3’
